# Evolving the Anthropocene: linking multi-level selection with long-term social–ecological change

**DOI:** 10.1007/s11625-017-0513-6

**Published:** 2017-11-23

**Authors:** Erle C. Ellis, Nicholas R. Magliocca, Chris J. Stevens, Dorian Q. Fuller

**Affiliations:** 1 Department of Geography and Environmental Systems, University of Maryland, Baltimore County, Baltimore, MD 21250 USA; 2 Department of Geography, University of Alabama, Tuscaloosa, AL 35487 USA; 3 Institute of Archaeology, University College London, London, WC1H 0PY UK

**Keywords:** Sociocultural niche construction (SNC), Agent-based modeling (ABM), Social–ecological systems (SES), The extended evolutionary synthesis (EES), Anthroecology, Archaeology

## Abstract

To what degree is cultural multi-level selection responsible for the rise of environmentally transformative human behaviors? And vice versa? From the clearing of vegetation using fire to the emergence of agriculture and beyond, human societies have increasingly sustained themselves through practices that enhance environmental productivity through ecosystem engineering. At the same time, human societies have increased in scale and complexity from mobile bands of hunter-gatherers to telecoupled world systems. We propose that these long-term changes are coupled through positive feedbacks among social and environmental changes, coevolved primarily through selection acting at the group level and above, and that this can be tested by combining archeological evidence with mechanistic experiments using an agent-based virtual laboratory (ABVL) approach. A more robust understanding of whether and how cultural multi-level selection couples human social change with environmental transformation may help in addressing the long-term sustainability challenges of the Anthropocene.

## Introduction

Humans, unlike any other species in Earth’s history, gained the capacity to transform an entire planet (Waters et al. 2016; Steffen et al. 2016; Ellis 2015). Anthroecology theory proposes that human societies gained this capacity through a long-term evolutionary process coupling increases in societal scales with increasingly intensive ecosystem engineering (Ellis 2015). This paper examines the role of cultural multi-level selection (CMLS) in shaping the long-term social–ecological changes that enabled human societies to scale up and transform Earth through its structuring effects on sociocultural niche construction (SNC), the alteration of sociocultural, ecological, or material patterns and processes by human individuals, groups, or populations through socially learned behaviors, exchange relations, and cooperative engineering in ways that confer heritable benefits and/or detriments to these individuals, groups, or populations (Ellis 2015).

Though contemporary scales and rates of anthropogenic environmental transformation are unprecedented, human societies began transforming Earth’s ecology thousands of years ago (Ruddiman et al. 2015; Kirch 2005; Boivin et al. 2016; Ellis et al. 2013b; Ellis 2015; Ellis et al. 2016). As Earth’s “ultimate ecosystem engineers”, humans have long used fire to clear land, propagated and domesticated plants and animals, tilled soils, built settlements and engaged in a wide range of other environment-modifying behaviors (Smith 2007b). Over time, human capacities to engineer ecosystems evolved to support larger and larger populations, producing ecological inheritances with both beneficial and harmful adaptive consequences through evolutionary processes of niche construction (Smith 2007a; Ellis 2015; Odling-Smee et al. 2003).

Humans are also Earth’s most social species, with an unrivaled capacity for social learning, accumulating cultural inheritances, culturally defined social relations (specialization, institutions, social identities), and dependence on non-kin exchange relationships, which together mark us as Earth’s first ultrasocial species (Richerson and Boyd 1998; Hill et al. 2009; Gowdy and Krall 2013, 2016). As human capacities for social learning increased, at least partly facilitated by the emergence of languages, cultural inheritances accumulated and cooperation within social groups became a major force shaping human evolution, driving one of Earth’s great evolutionary transitions: the rise of ever larger scales of human societies shaped increasingly by cultural selection at the group level and above, CMLS (Jablonka and Lamb 2006; Wilson 2010; Henrich 2015). Through CMLS, human societies evolved to become increasingly complex, specialized and hierarchical (Wilson 2012; Wilson and Wilson 2007; Henrich 2015) and cultural evolution became sociocultural evolution (Ellis 2015).

Human sociocultural evolution and niche construction are clearly linked. Over millennia, human societies accumulated an increasingly complex and potent suite of culturally inherited, socially learned and socially enacted practices for niche construction, such as domestication, livestock husbandry, and irrigation that have increased environmental productivity in support of human populations (Smith 2007a; Ellis et al. 2013b; Ellis 2015; Zeder 2016; Fuller and Lucas 2017). Even the most productive hunting and foraging strategies were capable of sustaining no more than a dozen hunter-gatherers on a single square kilometer of land (Ellis 2015). Through increasingly intensive agricultural practices, that same square kilometer of land might now be managed to sustain thousands in agricultural and industrial societies (Ellis et al. 2013b).

The niche construction practices of hunter-gatherer societies might ultimately have sustained populations of a few tens of millions at global scale, while agricultural societies have supported hundreds of millions for millennia and industrial societies have sustained billions for nearly a century (Ellis 2015). As human societies scaled up, their socially learned and socially enacted niche construction behaviors evolved into the “great force of nature” that is causing Earth’s transition to a new epoch of geologic time; the Anthropocene (Waters et al. 2016; Steffen et al. 2016; Ellis 2015; Turner II and McCandless 2004; Gowdy and Krall 2013, 2016). As a result of the ongoing evolution of human sociocultural niche construction in the Anthropocene, ecological change is social change, and social change is cultural change (Ellis 2015).

## Agriculture and urbanization: archeological evidence of regime shifts in social scale and niche construction

A growing body of archeological research documents empirically how human societies around the globe underwent fundamental shifts in ecosystem engineering, population density and social system complexity. The many regional records of sociocultural evolution also provide evidence of two major recurrent regime shifts in societal scale and niche construction. Archeologists have long referred to these transitions as the Neolithic, or agricultural, revolution and the urban revolution (Childe 1936; Hassan 1981). Agriculture was a turning point that brought about new species (domesticates), new ecologies (arable fields and pastoralism) and new socio-economies (sedentary communities based on storage and land-ownership). Sedentism and agriculture also emerged alongside increased investment in making material culture, from more elaborate and long-lasting buildings, to ceramics, the first textiles, and a wide range of art (Renfrew 2001; Hodder 2012). The setting of permanent villages and buildings, art and artefacts, provided central locations and mnemonics for the transmission of cultural inheritance and helped reinforce the emergence of larger social scales. The ultimate impacts of domestication and agriculture were realized with the next scaling up that occurred with urbanization, as larger concentrations of populations, including growing numbers of non-farming specialists and growing trade networks, were supported (Scott 2017). With the expansion of cities, longer supply chains of trade contributed to feeding the cities, while the intensity and range of material production also increased.

Plant and animal domestications underpinning the origins of agriculture occurred in parallel around 20 times globally, and despite differences, confirms the parallel adaptations on the part of crops to the human sociocultural niche (Fuller et al. 2014). The domestication process in cereals and other grains made these plants increasingly dependent on humans for seed dispersal, but also required increased human labor investment while increasing yields. In China, for example, millet and rice domestication took place along the Yellow and Yangtze rivers, respectively, between 9000 and 5000 years ago and over this period human populations grew more than exponentially, based on both the rapid increase in site number and site size (Stevens and Fuller 2017). In Western Asia, domestication was focused between 11,000 and 9000 years ago, and there too population expanded quickly (Fuller et al. 2014). Early agricultural villages had populations in the 100 s, although as the Neolithic progressed, some settlements comprising 1000 s of individuals emerged. Cultivation represented a new ecology that included small-scale intensive efforts to maintain and increase the productivity of land, evident through weed flora analyses on early Chinese rice (Weisskopf et al. 2015), and stable isotopes from archeological grains from the eastern Mediterranean (Styring et al. 2017). Thus, early farming scaled up labor invested per unit of land, the magnitude of environmental impacts and the size of social exchange networks.

Through urbanization, the first cities emerged with populations in 10,000 s, operating as centers of diverse communities, where some people took on specialized roles. These appeared in parts of Western Asia by 5500 years ago and central China by 4000 years ago. Cities drew in raw agricultural produce from the surrounding countryside, transformed it into added value commodities or redistributed agricultural calories to growing non-farming populations, which in turn produced a growing range of material commodities (metals, textiles, transport vessels, ornaments), and performed new administrative functions (Trigger 2003; Sherratt 2011). New forms of land use for orchards and vineyards added consumable commodities to the growing trade networks (Sherratt 1999). Cereal production played a critical role in underpinning early state formation. Cereal grains were storable, measurable and movable and fostered the development of writing, administrative systems, as well as increasingly hierarchical social systems (Steensberg 1989; Scott 2017). Urban demands for food grains lead to not only expanding the extent of agricultural land but also to more intensive ecosystem engineering of existing farmland through irrigation and field system creation. Major shifts in social structure also took place in terms of surplus being taxed, stored and redistributed through hierarchical non-kin based decision-making and expanded social networks (Scott 2017). Thus, while agriculture may have expanded, it also intensified, enabling growing populations to be supported from less farmland per capita.

## Which came first?

**Fig. 1:**
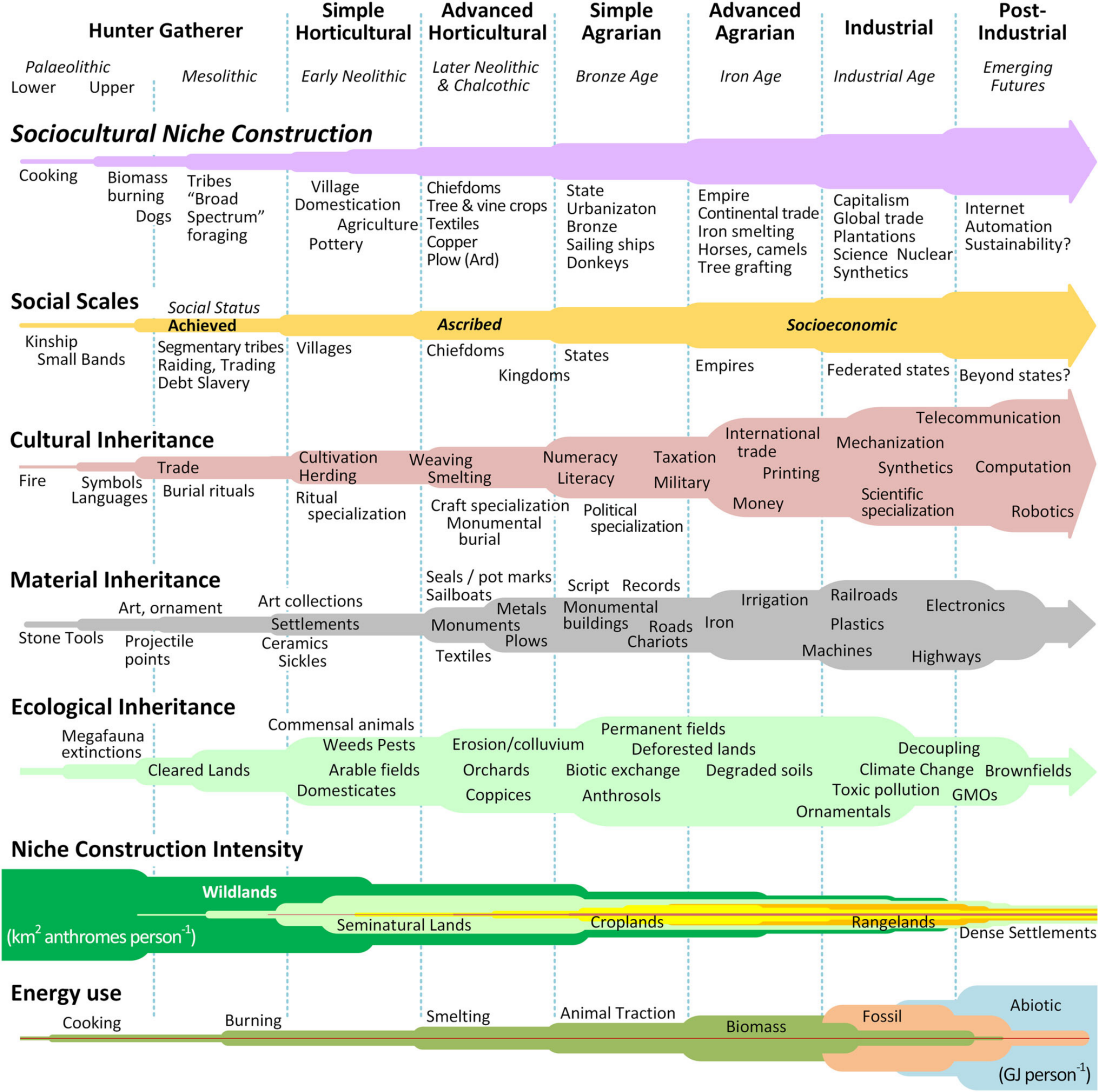
Major societal regime shifts in sociocultural niche construction (SNC; purple bar) compared in terms of societal types, archeological ages, scales of social structure (gold bar), and their cultural, ecological, material inheritances (relative heights of pink, gray and green bars). Niche construction intensity is represented in terms of anthrome area per capita (lower per capita areas indicates higher productivity in support of human populations) and relative per capita energy use (increasing per capita energy use also generally indicates more intensive ecosystem engineering). All Y axes indicate relative, not absolute, changes. Based on Fig. 3 in Ellis (2015)

Archeological, historical, and ethnographic evidence confirms that societal scales have increased in parallel with the intensity of sociocultural niche construction (Fig. 1). The productivity of land and resource management, population size, population density, societal complexity, and the amount of nonhuman energy used per capita are all positively correlated across societies over time (Turner II et al. 1977; Nolan and Lenski 2010; Chase-Dunn 2006; Ellis et al. 2013b; Ellis 2015; Hassan 1981; Trigger 2003). But did increasingly productive niche construction practices cause human societies to scale up, or was it the other way around? In assessing these long-term societal trends, it is crucial to recognize that, like biological evolution, these trends are neither linear, progressive, nor inevitable. Rather, the patterns of extant and past societies form a complex tree-like structure shaped by diversification, retrogression and extinction interwoven with horizontal cultural exchanges that have produced a “fabric” of human sociocultural evolution (Gray et al. 2010; Ellis 2015). Nevertheless, over the long-term, small and egalitarian mobile bands of hunter-gatherers came first, then more sedentary, specialized, and increasingly unequal agrarian and urbanizing societies of tens of thousands to millions and ultimately, the highly stratified and unequal, urban industrial world system of interacting societies that sustains billions today.

Larger scale societies have larger populations, but are also characterized by greater accumulations of cultural, ecological, and material inheritances, including the cultural practices, individual and group specializations, social institutions, exchange relationships, technologies, domesticated species, altered environments, and built infrastructure that have enabled them to sustain larger populations in increasingly human-altered environments (Ellis 2015). In other words, larger scale societies are defined as much by their complex and culturally shaped hierarchical modes of social organization as by their larger populations and more productive practices of ecosystem engineering. It is entirely plausible that the sociocultural evolution of larger scale societies was itself the driver of increasingly productive ecosystem engineering—not the other way around. Yet the coupling of societal scale with ecosystem engineering intensity is best explained by a cyclical process of reciprocal causation, in which each causes the other (Laland et al. 2015).

Multiple authors have proposed that societal scale and ecosystem engineering are coupled through a cyclic system of positive feedbacks: upscaling drives intensification and intensification drives upscaling (Ellis 2015; Nolan and Lenski 2010; Chase-Dunn 2006; Pfaffenberger 1992; Gowdy and Krall 2016). The classic model of this coupled system is based on direct positive feedbacks between population and food production; populations grow, increase demand for food, and societies respond by increasing the intensity of ecosystem engineering, producing more food, causing populations to grow further (Nolan and Lenski 2010; Chase-Dunn 2006; White 1943). In some models, productivity increases are facilitated by increasing rates of technological innovation (Smith and Marx 1994). In others, innovation rates stay the same, but the increasing demands of growing populations lead to increasing adoption of more productive pre-existing technologies, a process known as induced intensification (Boserup 1965; Ellis et al. 2013b; Turner II and Ali 1996). The latter model, in which societal pressures select for intensive ecosystem engineering practices, offers the prospect for an evolutionary theory coupling societal upscaling and niche construction.

## Evolving the Anthropocene: Is CMLS necessary?

Anthroecology theory proposes that the long-term trend towards larger scale societies with increasingly intensive ecosystem engineering is the result of a runaway evolutionary process of sociocultural niche construction (Ellis 2015). Runaway evolutionary processes were first described by Charles Darwin to explain the evolution of extravagant plumage and other costly, seemingly non-adaptive traits through a directional selection process in which female preference for, and male expression of, these traits increased together through a system of positive feedbacks (Fisher and Bennett 1930). Building on this framework, Laland, Rendell and others (2000; 2011) proposed that a process of runaway cultural niche construction might explain why, early in human evolution, cultural traits for ecosystem engineering (cultural niche construction) began evolving so rapidly that they overwhelmed rates of natural selection for genetic adaptations to environmental conditions.

Runaway cultural niche construction occurs when socially learned traits for ecosystem engineering cause environmental changes that select for additional cultural or genetic traits (Rendell et al. 2011; Laland and O’Brien 2012). Classic examples of runaway selection for genetic traits are increasing frequencies of lactose tolerance genes among pastoralists and malaria resistance genes in rainforest cultivating farmers whose practices increased mosquito populations (Rendell et al. 2011). Niche broadening, also known as the broad spectrum revolution, is a classic example of runaway selection for cultural traits, occurring widely across hunter gatherer societies when increasingly intensive hunting and foraging strategies deplete preferred wild species, requiring further cultural adaptation by social learning to utilize new species, leading to the sociocultural capacity to exploit an ever broader range of species and the capacity to sustain larger populations in the same ecosystem (Zeder 2012). Another example is soil tillage, which reduces soil fertility over time, requiring ever more intensive agricultural practices to compensate, such as manuring, intercropping, or multi-cropping (Matson et al. 1997; Harris and Fuller 2014). In all these examples, the net result of runaway cultural niche construction is human societies increasingly dependent on cultural practices of ecosystem engineering and resource use to sustain themselves. Thus, runaway cultural niche construction can help explain rapid co-evolutionary changes in human genetics and cultural niche construction at the population level. Yet, the role of increasing selection pressures at the group level and above in shaping changes in societal scale are not considered in this theory.

Is a CMLS framework needed to explain coupled long-term increases in societal scale and environmental transformation? On the one hand, this seems self-evident. It is hard to imagine how increasingly complex and hierarchical large-scale societies could evolve without a framework capable of understanding the formation and interaction of social groups and societies. A CMLS approach is clearly critical for explaining the evolution of larger scale societies and even small scale societies (Wilson and Sober 1994; Reyes-García et al. 2016; Gowdy and Krall 2016). Yet, it is still possible to imagine evolutionary models in which larger and/or denser human populations might select directly for more intensive ecosystem engineering practices without incorporating the multilevel structure of human societies or their evolutionary changes over time. Group selection might be needed to explain societal upscaling, but not to explain increasingly intensive practices of ecosystem engineering.

If we wish to test whether group selection is required to explain the long-term coupling of human societal upscaling with increasingly intensive niche construction, it will be necessary to simulate long-term social–ecological changes in populations with and without selection pressures acting at levels above the individual. Empirical data from archeologists, paleoecologists, ethnographers, and environmental historians confirm that regime shifts in social scale and niche construction have tended to occur together, including the Neolithic transition and the urban revolution. Nevertheless, these data cannot resolve the causal mechanisms of these coupled regime shifts: larger scale societies always include both larger populations and more complex and hierarchical social structures. Without the ability to experimentally decouple the size of human populations and their demands for increasingly intensive niche construction practices from changes in social capacities to enact larger scales of cooperative ecosystem engineering and more effective social systems to exchange their produce effectively within and across social groups and societies, there is no way to determine causal relations between human social scale and niche construction intensity.

## Testing runaway sociocultural niche construction

The central hypothesis of runaway sociocultural niche construction is that human societal scale and ecosystem engineering intensity increase together through a self-reinforcing system of positive evolutionary feedbacks. As societies scale up, their capacity to engineer more productive ecosystem increases through the accumulation of cultural practices (technologies, exchange systems) and increasing levels of cooperation and exchange among specialist individuals and groups with different expertise (e.g., toolmakers, breeders, traders). More productive strategies for ecosystem engineering, often requiring larger scales of cooperation among specialists, increase the production of food, fiber and other resources, which enable larger populations, increased per capita consumption, and most importantly, the production of surpluses that can be extracted for social exchange through trade and taxation. When ecosystem engineering increases land productivity, it can also release labor from food production, creating new opportunities for increasing levels of social specialization and hierarchical societal development that include urban populations far from sites of food production. Increasingly complex hierarchical societies have the social capacity to engage in increasingly productive ecosystem engineering, and by increasing ecosystem productivity, they create the conditions necessary for the further evolution of social complexity in support of increasing societal scales.

The basic principles of runaway sociocultural niche construction can be expressed through four related hypotheses: (1) larger scale societies cannot sustain themselves without more intensive systems of food production, (2) more intensive food production systems are not possible without more specialized and increasingly cooperative societies, (3) neither can evolve independent of the other, and (4) positive feedbacks between societal upscaling and ecosystem engineering productivity can drive major, coupled, long-term increases in societal scale and environmental transformation. To test these hypotheses, it will be necessary to build a model capable of simulating human societal upscaling coupled with ecological system dynamics to simulate the intensification of ecosystem engineering across landscapes. Such a model must include selection processes acting on individual human agents and their cultural traits associated with ecosystem engineering, resource extraction, and exchange with other agents, both kin and non-kin, within and across social groups and societies. Similarly, such a model must be capable of generating emergent, self-organized social groups, selection among groups, and dynamic selection pressures on the cultural traits defining individual, within group, and across group behaviors. Finally, to close the positive feedback loop, ecological consequences of ecosystem engineering, including environmental degradation, productivity enhancement, and their interactions with environmental heterogeneity and stochasticity also need to be simulated. All of these processes would need to be modeled in such a way that social processes and selection at group and societal levels, and their capacities to enact increasingly productive niche construction regimes, could be turned on, turned off, or set to various levels, to test the roles and relative importance of each in producing runaway sociocultural niche construction over many generations across increasingly large and complex agent populations in plausible social–ecological scenarios.

Taken together, the requirements of such a model are clearly daunting. Nevertheless, there are clear prospects for building models capable of testing the basic hypotheses of runaway sociocultural niche construction. One of these prospects is an agent-based virtual laboratory (ABVL) approach employing a ‘generative social science’ mode of inquiry focused on developing and testing general theory on social–ecological interactions; ‘growing’ human societies and their adaptations to and of their environments from the bottom-up (Epstein 1999; Magliocca and Ellis 2016; Barceló and Del Castillo 2016). The ABVL approach couples agent-based models (ABM) simulating human individual and social behaviors with environmental models to conduct evolutionary experiments in which alternative, candidate processes governing these behaviors can be experimentally manipulated to test their emergent social, ecological, and landscape patterns and dynamics against empirical evidence (Magliocca and Ellis 2016; Barton et al. 2016).

## An agent-based virtual laboratory (ABVL) approach

To move forward with an ABVL approach, a number of challenges are clear. The first is the need to assemble suitably detailed and reliable long-term spatially explicit datasets of social–ecological change across regions to enable model parameterization and/or validation for hypothesis testing. While empirical reconstructions of long-term cultural, social and environmental change will always be incomplete, such datasets are increasingly available through the efforts of archeologists, geographers, environmental historians and other scholars (Zeder 2016; Turchin et al. 2015; Ellis et al. 2013a; Barceló and Florencia 2016; Boivin et al. 2016). From a model design and utilization point of view, there are even greater challenges.

Efforts to develop ABMs to test theory on the mechanisms of social–ecological change are beginning to bear fruit (Waring et al. 2017; Verburg et al. 2016; Janssen and Hill 2016; Janssen et al. 2007; Heckbert et al. 2016). ABM’s developed using a CMLS framework have simulated rich representations of emergent cooperative behavior, economic institutions, group selection, and cultural evolution within stylized environmental settings, demonstrating linkages among environmental conditions and individual and group behaviors, norms, institutions, and sustainable resource use regimes; cultural group selection has already been shown to facilitate sustainable societal behaviors (Waring et al. 2017, 2015; Schill et al. 2016). Generalized ABMs of human–environment interactions, such as those developed as part of the MedLab project, have also produced insights into the mechanisms of long-term social–ecological change by pairing behaviorally simple ABMs with relatively rich landscape evolution models across a variety of biophysical settings in a form enabling successful comparisons archeological and paleoecological evidence (Barton et al. 2016).

Even with these advances, combining rich representations of both sociocultural and ecological processes and simulating their evolutionary feedbacks and emergent dynamics in realistic simulated landscapes over long time periods in a form capable of testing anthroecology theory against empirical evidence remains a major challenge and direction for future work, as described by Magliocca and Ellis (2016). Model design choices, such as the number of agents and spatial and temporal scales of simulation, must align with available evidence. For example, some processes, such as societal decline, might not have a discernible signature in the archeological record (Alroy 2001). Similarly, simulation of individual agents or households might be made consistent with theories of optimal foraging or labor-minimizing cultivation strategies, for example, but additional assumptions will be needed to translate the activities of such agents into evidence comparable with that available in the archeological record. Environmental dynamics must be sufficiently realistic to represent influences on agent decision-making processes—such as agricultural intensification or relocation in response to declines in agricultural productivity due to soil degradation (e.g., (Magliocca et al. 2013)—but no more. Which environmental dynamics to simulate explicitly, and which to abstract or simplify, will depend on the empirical evidence of environmental changes that can be estimated or reconstructed. For example, to simulate the introduction, transmission and inheritance of ecological, cultural and material innovations (e.g., domesticates, cultivation practices, institutions, and physical infrastructures, such as irrigation systems), it may be more useful to simplify and abstract these into functional types, such as intensive cropping (e.g., irrigated rice) versus extensive cropping (e.g., shifting cultivation based on cassava). Such abstraction allows the simulation of important dynamics in the face of limited or inconsistent data while maintaining model generality over space and time.

Even greater challenges stem from need to confront simulated processes themselves with empirical evidence at appropriate levels to ensure that these are realistically represented (i.e., structural validation; (Brown et al. 2005; Grimm et al. 2005; Latombe et al. 2011). Specifically, processes involved in the formation and dynamics of social structures are essential for simulating social–ecological change, but difficult to observe in archeological evidence. Such processes include: demographics at the household level, social groups, and societies (Barton et al. 2016), group formation and competition, including the role of warfare (Turchin et al. 2013), and the role and scale dependence of groups and social networks in facilitating shifts in social capacities for cultural transmission and accumulation (Powell et al. 2009). Clearly, there is much hard work ahead on the road towards an experimental framework capable of investigating the evolutionary mechanisms behind long-term social–ecological change.

## A way forward

Anthroecology theory proposes that human societies gained the capacity to transform a planet, without intending to, through a runaway evolutionary process of sociocultural niche construction which caused societal upscaling and niche construction intensity to increase together. If these trends continue into the future, the results would likely be no better than they have been in the past: the generation of ever larger-scale societies, with ever larger populations continuing to shift the Earth system towards a hotter, more polluted, less biodiverse and less wild state. While human populations appear to be leveling off as a result of increasing development and urbanization, and livelihoods and longevity continue to improve, billions more are expected no matter how rapidly growth rates are reduced (Bradshaw and Brook 2014).

Archeological evidence confirms that larger scale societies and more intensive niche construction practices evolved in parallel, but cannot determine whether these are mechanistically coupled through positive feedbacks. Is it possible for the intensity of sociocultural niche construction to increase even faster than growth in populations and per capita environmental demands? In other words, can the environmental demands of human societies shrink while populations continue to grow? There is some evidence that this may have occurred at times in the past and is in fact occurring now, as global agricultural land use has generally been growing more slowly than populations in recent decades, increasing food available per capita (FAO 2017; Ellis et al. 2013b). Either way, without long-term increases in land use intensification, it is likely that human demands for land will cause habitat and biodiversity losses to continue (Dinerstein et al. 2017).

Even for the conditions of the deep past, when societies were smaller and less complex, the development of experimental approaches fully capable of testing mechanistic hypotheses on runaway sociocultural niche construction remain at an early stage of development. Achieving this capacity for contemporary societies will require overcoming serious technical, theoretical and empirical challenges. Nevertheless, the ABVL approach has the potential to investigate key questions of sustainability science. Is human sociocultural evolution sustainable over the long term? How will sociocultural evolution shape future trajectories of social and environmental change? How can these evolutionary processes be guided towards better outcomes for both humanity and nonhuman nature? By developing experimental approaches capable of testing hypotheses on the evolution of societies and the sociocultural niche construction regimes that sustain them, critical knowledge may be gained towards understanding and influencing societal transformation of Earth towards more sustainable and desirable futures.
